# Peptide Dose and/or Structure in Vaccines as a Determinant of T Cell Responses

**DOI:** 10.3390/vaccines2030537

**Published:** 2014-07-02

**Authors:** Graham R. Leggatt

**Affiliations:** The University of Queensland Diamantina Institute, Translational Research Institute, 37 Kent Street, Woolloongabba, Brisbane QLD 4102, Australia; E-Mail: g.leggatt@uq.edu.au; Tel.: +61-7-3443-6961; Fax: +61-7-3443-6966

**Keywords:** vaccination, T cell avidity, altered peptide ligands, peptide dose, peptide structure, peptide vaccines, functional avidity

## Abstract

While T cells recognise the complex of peptide and major histocompatibility complex (MHC) at the cell surface, changes in the dose and/or structure of the peptide component can have profound effects on T cell activation and function. In addition, the repertoire of T cells capable of responding to any given peptide is variable, but broader than a single clone. Consequently, peptide parameters that affect the interaction between T cells and peptide/MHC have been shown to select particular T cell clones for expansion and this impacts on clearance of disease. T cells with high functional avidity are selected on low doses of peptide, while low avidity T cells are favoured in high peptide concentrations. Altering the structure of the peptide ligand can also influence the selection and function of peptide-specific T cell clones. In this review, we will explore the evidence that the choice of peptide dose or the structure of the peptide are critical parameters in an effective vaccine designed to activate T cells.

## 1. Introduction

**Figure 1:**
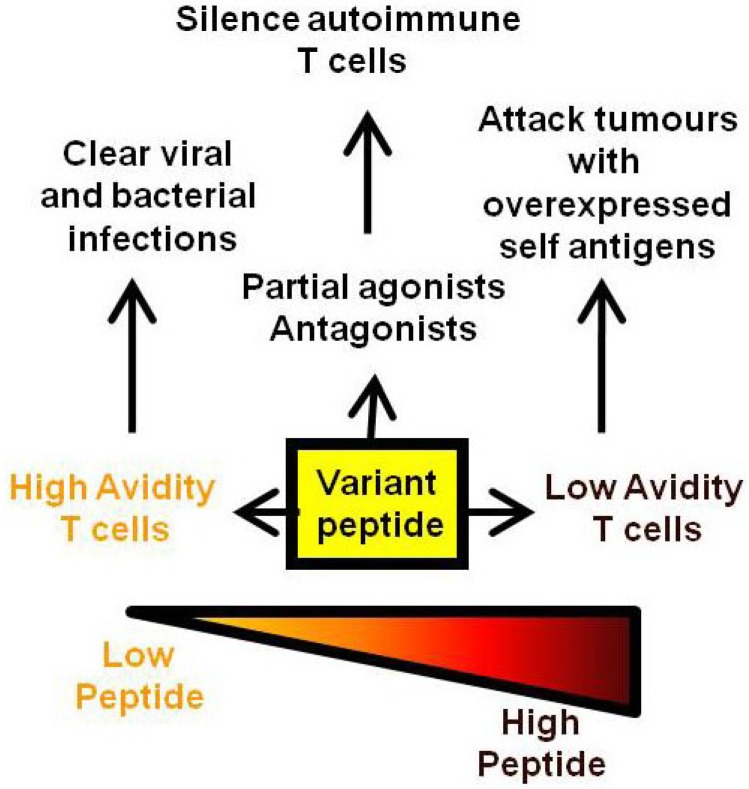
Summary of the effects of variant, low and high dose peptide on effector T cell responses.

Many of the traditional vaccines against infectious disease have involved the use of attenuated live or killed organisms with excellent results such as the elimination of smallpox [1,2]. The use of whole organisms allows for the display of multiple antigenic determinants with a subset of these antigens often dominating the immune response. Given the efficacy of these established vaccines, it appears that nature provides these selected epitopes in the context of the whole organism at the appropriate dose and timing to allow efficient T and B cell priming. In particular, many of these vaccines rely on the development of neutralising antibody which may have flexible requirements for epitope dose *in vivo* above some minimum threshold [3]. In contrast, infectious agents for which no vaccine currently exists, such as HIV, are likely to have a greater dependence on T cell mediated clearance [4,5]. The activation of naive T cells in lymphoid organs is most efficiently achieved by dendritic cells (DCs). The engagement of pathogen recognition receptors (such as Toll-like receptors or TLRs) on the surface of tissue resident DCs leads to their maturation and migration to lymphoid organs. Here, the matured DCs present MHC/peptide complexes along with co-stimulatory ligands and cytokines to activate naive T cells and enable their conversion into effector or memory T cells. An efficient and appropriate conversion of naive T cells into effector/memory T cells is at the heart of current vaccine development. An early study showed that ovalbumin peptide delivered intravenously was able to tolerise peptide-specific CD4 T cells while the same peptide administered in complete Freund’s adjuvant via a subcutaneous site generated peptide-specific memory T cells [6]. This highlights the importance of peptide context in vaccine design and many vaccine studies are now focused on combining peptide with appropriate co-stimulatory molecules and a pro-inflammatory cytokine environment through the use of different adjuvants, delivery vectors and immunisation routes [7,8,9,10,11]. It is unlikely that any one combination of these variables will lead to a “universal” vaccine platform given the diversity of infection pathways utilized by pathogens. Aside from important co-stimulatory/cytokine considerations in vaccine development, there is also a central role for peptide in dictating the TCR signaling which leads to T cell proliferation and effector function. Several of the infectious diseases that require vaccine targeting are chronic infections that induce high or chronic loads of antigen. Under these circumstances, T cells can become anergised, exhausted or die of apoptosis [12,13,14]. Early *in vitro* experiments measuring the T cell response (likely CD4 T cells) to antigen have shown that too much antigen results in inhibition of the T cell proliferative response [15]. A similar high dose inhibition has also been documented for CD8 T cells [16]. So, while the effector function, measured as cytotoxicity or cytokine release, does not generally diminish with increasing peptide concentration, the ability of the T cell clone to divide and expand can be compromised. If mirrored *in vivo*, this is likely to affect the available T cell repertoire and subsequent pathogen clearance. Changing the peptide structure, as occurs in the natural viral variants arising during influenza infection, will also alter the responding repertoire of T cells either to allow immune escape or to establish a cross-reactive immune response [17]. This review will focus on studies which have examined the effects of peptide dose and/or structure on the repertoire of responding T cells and the outcomes for protection against disease (summarized in Figure 1).

## 2. Peptide Dose

Unlike whole organism vaccines, protective peptide vaccines are reliant on the correct choice of peptide within complex proteins and a decision on the amount of peptide to be administered. With the latter, it raises the question of whether too little or too much peptide would impact on the effectiveness of the immune response. Very early studies reported on the phenomenon of high and low zone tolerance after immunization with different doses of antigen [18,19]. Injection of cyanogen bromide digested flagellin into rats resulted in high and low doses of flagellin antigen being inhibitory to antibody production and delayed type hypersensitivity responses [19]. These studies suggested that for any given protein/peptide an optimal dose of immunogen exists and that this dose may need to be empirically determined for each vaccine construct. Consequently, early clinical trials often involve dose escalation studies of a new vaccine [20,21,22]. In many of these studies, the maximum tolerated dose is chosen for further trials given that mean antibody titres are often seen to increase in proportion to the dose of administered antigen [20]. While the quantity of antibody achieved after immunisation is important, it is now recognised that the quality of the antibody response as defined by immunoglobulin isotype and affinity for antigen is also a determinant of neutralising capability [3,23]. Fewer studies have focused on T cell responses after dose escalation, although in parallel with the antibody studies, it is generally the numbers of induced, antigen-specific T cells that are reported rather than the quality of the T cell response [24]. Evidence for dose escalation impacting on T cell response comes from studies where increasing the dose of influenza virus during primary infection in mice altered the immunodominance hierarchy of individual peptides and the repertoire of responding T cells [25].

During the course of a natural immune response, antigen-experienced T cell populations acquire a greater sensitivity to peptide/MHC signals than naive T cells in a process termed “avidity maturation” [26,27,28]. This can occur through selective outgrowth of the highest affinity T cell clones over time but may also be independent of T cell receptor (TCR) affinity and involve changes to the intracellular signaling machinery or clustering of TCR into lipid rafts for more efficient signaling [29]. Using lymphocytic choriomeningitis virus (LCMV) infection of mice, it was shown that T cell avidity increased over time in monoclonal CD8 T cell populations (fixed TCR affinity) and was dependent on changes to signal transduction within the cell [27]. Avidity maturation of T cells may be a consequence of diminishing antigen during resolution of infection and provide the memory T cell pool with enhanced ability to respond upon secondary infection [30]. It remains to be determined if sequential reduction in peptide/antigen dose over multiple vaccinations would promote avidity maturation and a highly sensitive memory T cell pool. Avidity maturation is not always associated with a positive outcome for the immune system. In instances such as type 1 diabetes the avidity maturation of pancreatic beta cell-specific CD8 T cells associates with progression toward diabetes [26]. Multiple injections of soluble peptide was able to delete the diabetic T cells in this situation thus providing a therapeutic tool to ablate avidity maturation. Care must also be taken in using very low doses of agonist peptide in vaccines to stimulate CD4 T cells since suboptimal peptide can lead to the development of FoxP3-expressing T cells with potential regulatory function [31]. FoxP3-expressing T cells are generally induced by agonist peptide doses that fall below the threshold required to activate effector/memory T cells.

The quality of a T cell response can be measured by parameters such as the functional avidity (effector function over titrated doses of peptide) and range of effector function. In previously published work, we have utilized high and low doses of a minimal cytotoxic T lymphocyte (CTL) peptide during tissue culture to generate CD8 T cells polarized toward high and low avidity populations [32]. Consequently, high avidity T cells selectively grew under low peptide conditions while low avidity T cells were favoured by high peptide concentrations. In this same study, high avidity T cells were shown to be more efficacious than low avidity T cells in their ability to reduce viral infection when transferred into immunodeficient mice (RAG1^−/−^) that were subsequently infected with antigen-expressing vaccinia virus. The observation of improved anti-viral activity amongst antigen-specific, high avidity T cells has been replicated for many viral infections [33,34,35]. Our studies also demonstrated that low avidity T cells dominated the cultures grown on high levels of peptide because this peptide concentration resulted in apoptosis of high avidity T cell clones via a TNF-α dependent mechanism [16,36,37]. The selective deletion of high avidity T cells has also been shown *in vivo* for CD4 T cells specific to Salmonella antigens [38] and CD8 T cells in chronic myelogenous leukemia (CML) [39]. Low avidity T cells have been shown to prevent the outgrowth of murine tumours over expressing self antigen (*i.e.*, high levels of peptide/MHC) while ignoring lower levels of the same self antigen on non-cancerous tissue [40]. The control of functional avidity within T cells can be influenced not only by the peptide level presented on MHC, but also by the number of engaged T cell receptors [41], the CD8 co-receptor [42,43], co-stimulation [44,45], cytokines such as IL-15 [46] and intracellular signaling events [47]. While existing data would be consistent with outgrowth of subsets of T cells with fixed avidity, other studies have suggested that individual T cells may give rise to high and low avidity effector T cells suggesting a degree of cellular adaptation [48]. The generation of high avidity CD8 T cells *in vitro* has been used by several groups to generate T cells for adoptive immunotherapy against tumours and viruses [49,50]. While it is clear *in vitro* that peptide concentration can skew the avidity of T cells, evidence for peptide dose affecting repertoires *in vivo* is also apparent. One study has shown that the ratio of high to low avidity T cells varies during the course of an immune response to recombinant vaccinia [51]. The ability to alter the avidity of T cell populations may be a property of the antigen-presenting cell. A study examining different amounts of peptide loaded onto mature dendritic cells demonstrated that high avidity T cells were generated at all peptide doses [52]. In the same study, non-professional antigen presenting cells were able to stimulate CD8 T cells of differing functional avidity based on the concentration of peptide loaded onto the cells. This suggests that high concentrations of peptide pulsed on to DCs is incapable of deleting high avidity T cells during the primary response and would be consistent with these DCs priming the full spectrum of T cell avidity for this antigen. It appears that the peptide dose *in vivo* may have a greater influence on skewing T cell avidity of effector/memory populations when compared with naive T cell activation. A second study using peptide-pulsed DCs also concluded that the avidity of the primary T cell response is not affected by peptide dose but subsequent recall or secondary responses were affected by peptide density on DCs [53]. Increasing the peptide dose in these studies increased the number of antigen-specific T cells during the primary response, a finding consistent with other studies in the literature [54,55]. Overall, it appears that peptide loaded DCs prime the full spectrum of antigen-specific T cells while selection of low and high avidity responses is a property of non-professional antigen presenting cells or recall responses. 

## 3. Peptide Structure

A second parameter driving the quality and quantity of the T cell response is peptide structure. Substituting amino acids within a T cell epitope can alter both the binding to MHC and/or T cell receptor leading to changes in TCR signaling which cannot be achieved by simply altering peptide dose. For instance, our own studies have shown that the outgrowth of high and low avidity T cells cannot always be achieved by varying the peptide dose. Using a human papillomavirus (HPV)-derived peptide, we have shown that high concentrations of peptide *in vitro* did not allow the growth of any antigen-specific T cells while low levels of peptide expanded the expected high avidity T cells [56]. The lowest avidity T cells for the wild type peptide could only be grown if a variant peptide with a single amino acid substitution was employed. The variant peptide binds more tightly to MHC I molecules and elicits CD8 T cells that are cross-reactive with wild type, HPV peptide [56]. We have speculated that the more stable MHC binding of variant peptide enables longer interaction with the TCR and subsequent development of the low avidity response. More broadly this suggests that variant peptides may have unique properties not found in the wild type peptide resulting in a broader repertoire of T cells after vaccination. Consistent with this idea, prime boost vaccination with different concentrations of wild type peptide failed to shift the functional avidity of the HPV peptide-specific CD8 T cell response *in vivo* [57]. In contrast, in the same study, priming with wild type peptide followed by boost with a high dose of the variant peptide led to a predominance of low avidity T cells specific for wild type peptide. Variant peptides can also favour the development of high avidity T cell responses as a study in melanoma used a low concentration of variant peptides to stimulate a high avidity CD8 T cell population capable of delaying the growth of B16 melanoma after adoptive transfer of T cells into the tumour bearing mice [58]. Importantly, immunisation with the variant peptides must induce T cell reactivity to the wild type peptide to be effective [59]. In another study using the CD4 T cell epitopes, moth cytochrome C (MCC) 88–103 and a variant peptide MCC(102S), vaccination with the variant peptide was able to induce high affinity, “resident” follicular T helper cells which contributed to B cell immunity [60]. 

The numbers of responding T cells to wild type tumour peptides can often be increased by immunisation with altered peptides that bind with higher affinity to MHC molecules [61]. Variant or altered peptide ligands have also been pulsed onto dendritic cells which are then infused into the patient [62]. The resulting T cells in this study were able to recognise tumour cells expressing the wild type epitope. The use of variant peptides to elicit CD8 T cells in tumour systems is common when the tumour antigen is a self protein that has resulted in deletion of high avidity T cell clones in the thymus. In one melanoma study, a variant peptide based on the sequence of human gp100 led to induction of T cells cross-reactive with mouse gp100 and capable of destroying B16 melanoma cells in tumour-bearing mice [63].

Variant peptides not only select cells of different avidity but can change the function of the T cells. In murine experimental allergic encephalomyelitis (EAE; a model for multiple sclerosis), altered peptides containing single amino acid substitutions were utilized to prevent disease development through switching T cell cytokine production toward a Th2 phenotype [64]. However, the current use of altered peptide ligands in the clinic for multiple sclerosis has been hampered by unexpected cross-reactivities and adverse reactions resulting in the termination of two clinical trials [65,66]. The polarization toward CD4^+^ Th1 and Th2 immune responses has been explored using variant peptides with the demonstration that peptide binding affinity to MHC and the duration of TCR interaction both affect the skewing of the CD4 T cell response [67,68]. Furthermore, the physiochemical properties of peptide residues such as hydrophobicity may also contribute toward Th1 *versus* Th2 polarization [69] although the ability to universally predict the activity of a peptide based on sequence or other intrinsic properties remains a challenge. Partial agonists are variant peptides which elicit a subset of the T cell functions generated by the wild type peptide [70,71]. Using a panel of gp100 substituted peptides, an altered peptide capable of inducing cytotoxic function but limited cytokine secretion from responding T cells was identified [72]. Consequently, APLs which alter the function of T cells may be useful in autoimmune disease settings or diseases dependent on a Th1/Th2 balance where silencing of selected T cell functions could be beneficial. Altered pathogen peptides which induce weak TCR interactions compared with the wild type pathogen peptide have been used in “thymic vaccination” to encourage positive selection of T cell clones specific for pathogen in the periphery [73,74]. This approach may be relevant when precursor frequencies of pathogen-specific T cells in the periphery are limited. Whether this approach might also result in negative selection or thymic deletion of T cell clones potentially useful in other pathogen responses requires careful consideration. 

Variant peptides can also have antagonistic properties resulting in the silencing of productive TCR signaling from wild type peptide. Antagonist peptides may be particularly useful in shutting down autoimmune T cells in diseases such as EAE [75], arthritis [76], celiac disease [77,78] and experimental myasthenia gravis [79]. Antagonist peptides can also operate at the level of MHC binding with one study showing that the development of EAE could be reduced with antagonist peptides that bound with high affinity to MHC II molecules [80]. Overall, the diversity of T cell responses elicited by altered peptide ligands suggests that peptide manipulation of the immune response is possible through vaccination. A limitation of this approach is that T cell responses to variant peptides still need to be empirically determined for each peptide immunogen.

## 4. Conclusions

Both peptide dose and peptide structure have a clear impact on the T cell response both *in vitro* and after immunization *in vivo*. Vaccination strategies need to consider the balance between generating large numbers of responding T cells and ensuring the appropriate quality of the resulting T cell population. High levels of peptide presented by MHC on dendritic cells does not appear to bias the functional avidity of the T cell repertoire and would seem to be an optimal strategy for priming large numbers of high avidity T cells to combat viral or bacterial infection. Alternatively, careful consideration of peptide dose would be needed if peptide is directed toward non-professional antigen presenting cells during primary vaccination or if peptide is used for subsequent vaccinations. Under these conditions, high peptide dose will favour lower avidity T cells while low dose peptide will lead to the generation of high avidity T cells. While some predictions can be made about the generation of T cells when varying peptide dose, multiple outcomes are possible for the T cell when the structure of the peptide is changed. Altered peptide ligands can be agonists, partial agonists or antagonists of the T cell response although this is often difficult to predict from the amino acid sequence. Rigorous testing is still required with altered peptide ligands to avoid activation of autoimmune T cells and ensure clinical safety. Perhaps future structural analysis of different TCR interactions with altered peptide ligand will assist the development of tools to more carefully predict cross-reactivities and improve patient outcomes. Consequently, in addition to choosing peptides which bind to relevant MHC molecules, vaccines should be designed to optimize the peptide dose and/or consider changes to peptide structure to promote both a quantitatively and qualitatively superior T cell response. 
